# Increasing medical student confidence in gender and sexual health through a student-initiated lecture series

**DOI:** 10.30476/JAMP.2021.90099.1398

**Published:** 2021-10

**Authors:** JASMIN MAHABAMUNUGE, KAYLA MOREL, JOHN BUDROW, INNES TOUNKEL, CASSIDY HART, CAMILLE BRISKIN, MADISON KASOFF, SARAH SPIEGEL, DONALD RISUCCI, JENNIFER KOESTLER

**Affiliations:** 1 New York Medical College, Department of Medical Education, School of Medicine, Valhalla, NY, USA; # These authors contributed equally

**Keywords:** Medical students, Curriculum, Gender and sexual minorities, Sexual health, Sexuality, Gender identity

## Abstract

**Introduction:**

Medical students self-report insufficient training in topics of gender and sexuality in medicine, which may ultimately lead to negative health outcomes in patients for whom they will provide care. This study aims to identify whether a student-initiated lecture series on topics related to gender and sexual health leads to greater student comfort with discussing topics related to diverse sexual content.

**Methods:**

Medical students matriculated during two consecutive academic years were invited to participate in the lecture series. Investigators administered anonymous pre- and post-series surveys (n=152 and 105 respondents, respectively) using google forms. Respondents rated their comfort levels discussing relevant topics and provided narrative feedback concerning strengths and areas for improvement of the lecture series. Overlaps between the 95% confidence intervals around pre- and post-series percentage of students comfortable/very comfortable discussing each topic were examined to compare pre- vs post-series comfort ratings. Narrative comments were reviewed for thematic feedback.

**Results:**

105 medical students completed the lecture series, with 80% identifying as female. Self-assessed comfort levels across all seminar topics were greater in post- versus pre-lecture series surveys with the following topics showing the biggest differences (percentage of students “somewhat” or “very” comfortable [95% confidence intervals]: discussing sexuality with gender (68%[59-77] vs. 29%[22-36]) and sexual minority patients (84%[77-91] vs. 49%[41-57]), HIV prevention counseling (70%[61-78] vs. 20% [20-34]), identifying female genital cutting (44% [34-53] vs. 11%[6-16]), and discussing intimate partner violence (65%[55-74] vs. 33%[25-40]). Qualitative analysis indicated respondents found the lectures to be effective and believed they should be integrated into the required medical school curriculum.

**Conclusion:**

Our student-initiated lecture series was associated with greater student comfort discussing topics related to gender and sexuality with patients. This framework represents a useful method to address gaps in medical education and has the potential to improve health outcomes in multiple populations.

## Introduction

National surveys of medical students reveal students perceive that they receive insufficient training on gender and sexual health topics in medical school ( 1- 5 ). Data from a 2008 survey of U.S. medical schools reports that schools provide between three and ten hours of training in gender and sexual health across their entire curriculum ( 1 ). This amount of training may be insufficient to adequately train medical students to address patient needs, and some authors suggest that more effort be made to incorporate topics of gender and sexuality in medicine into medical student curricula ( 4 , 5 ). 

The relative lack of formal education on sexual health may explain why medical students report feeling underprepared to effectively address their patients’ sexual health ( 1 , 2 ), and why few physicians routinely ask patients about sexual well-being ( 3 , 6 ). Previous studies have demonstrated an increase in student comfort addressing these topics with patients when they received formal education on these subjects ( 1 , 7 ). Although some medical schools have attempted to increase the sexual health content in their curricula ( 2 , 8 ), diverse approaches to revise and supplement medical school curricula are still needed ( 4 ). Some authors suggest that in order to be successful, novel curricular changes should approach sexual health topics through an intersectional lens, and address sexual health across multiple life stages, and in racially and ethnically diverse populations ( 4 , 9 ). Other research suggests that informal, student-initiated curricula may be another vehicle to effectively influence curriculum change in this area ( 10 ). 

While medical school curricula have been shown to be broadly lacking in a variety of sexual health topics, studies have demonstrated that content related to gender and sexual minorities (GSM) is especially limited ( 5 ). In a study by Obeldin-Maliver, et al, one third of U.S. medical schools do not require any curriculum content specific to GSM ( 11 ). This finding may be linked to a perception of a lack of competence in treating GSM patients among medical students, which is compounded by the urgent health care needs of these populations. Specifically, in one study, 70% of gender minorities and over 50% of sexual minorities report that they have experienced discrimination while seeking healthcare ( 5 ). Other studies have demonstrated that discrimination contributes to healthcare avoidance and subsequent health disparities in GSM patient populations, including increased rates of cardiovascular conditions, psychiatric illnesses, and lifestyle comorbidities ( 12 , 13 ).

Additional training gaps exist with regard to education in intimate partner violence ( 7 ), sexuality of older adults and individuals with disabilities ( 4 ), and the inclusivity of discussions about contraception, abortion education, and prevention of sexually transmitted infections (STI) ( 3 , 4 ). In fact, only 75% of primary care physicians feel comfortable talking about sexual health and an even smaller 28% of primary care physicians feel comfortable prescribing Pre-exposure Prophylaxis (PrEP) ( 14 ). These findings support that increased attention to sexual health training during early medical education is needed to address these disparities. 

The goal of this study was to evaluate the effectiveness of a student-initiated lecture series to improve medical student comfort with diverse sexual health content. It also informs best practices with regard to curriculum implementation in this subject area. 

## Methods

### Study Design

New York Medical College School of Medicine (NYMC SOM), located in Valhalla, New York, is one of the nation’s oldest private health sciences universities (est. 1860). The SOM provides a comprehensive educational program whose goal is to develop well-rounded medical students who will become resilient, compassionate, and skilled physicians. The SOM is proud of its strong foundational science education, diverse affiliated clinical training sites, and the commitment of its faculty and administration to medical student education. 

All medical students matriculated in the SOM were invited to participate in a ‘Gender and Sexuality in Medicine’ seminar series via email during the 2018-2019 (n=847) and 2019-2020 (n=862) academic years. Attendance for the series was voluntary. Students who attended at least five lectures were recognized with a “certificate of completion.” Program assessment was accomplished using a pre-test/post-test design to collect student demographic characteristics and student comfort applying seminar material. 

Students who conveyed interest in the seminar series were invited to complete voluntary, anonymous pre-lecture series (n=152) and post-lecture series (n=105) surveys via google forms. Only students who attended five or more seminars were invited to complete the post lecture survey. These surveys collected student demographic characteristics and assessed student comfort applying seminar material in a variety of different ways: discussing topics presented with patients and counseling patients on lecture information (see appendix for complete list). All responses were collected with a 5-point rating scale as follows: “1=very uncomfortable,” “2=somewhat uncomfortable,” “3=neutral,” “4=somewhat comfortable,” and “5=very comfortable.” Additional questions on post-lecture surveys elicited qualitative feedback for the lecture series by asking two open ended questions: “please identify what you consider to be the strengths of the seminar series,” and “please identify what areas of the seminar series could be improved.” Questions were adapted from previously published surveys to assess sexual health education amongst medical students ( 15) .The New York Medical College institutional review board reviewed and deemed this study protocol (#12850) exempt. 

### Analysis

Data were analyzed by computing descriptive statistics to examine the demographic characteristics of the students who completed the pre and post-lecture series surveys, the attendance of each lecture in the series and the distribution of self-reported student comfort with lecture material. For each item on both the pre- and post-series surveys, we computed the percentage of students who were “somewhat” or “very” comfortable and the associated 95% confidence intervals. The extent of overlap between pre- and post-series confidence intervals was examined to determine whether the percentage of students expressing comfort was higher in post-series vs. pre-series responses. We do not report p-values as respondent questionnaires were anonymous and individual responses were not linked between pre and post surveys. Investigators conducted all analyses using the Statistical Package for the Social Sciences (SPSS; IBM version 24). 

Qualitative responses were also evaluated to identify significant themes. Investigators initially reviewed this information independently and subsequently met as a group to resolve discrepancies in interpretations to reach consensus and final theme interpretation. Qualitative data was reviewed until no new themes emerged from the analysis. Illustrative quotes were collected to represent all salient themes.

## Results

The lecture series included fourteen lectures presented by content area experts, including clinicians, patients, and community stakeholders. Lecture topics included intimate partner violence, STIs and stigma, puberty suppression in transgender children, contraception and family planning, female genital cutting, and mental health in GSM patients (Table 1). The topics presented were chosen by student leaders from various advocacy and specialty groups across campus including but not limited to: LGBTQ Advocacy in Medicine, Obstetrics and Gynecology Interest Group, and Medical Students for Choice. Subject matter was chosen to address identified gaps in the required medical curriculum, as well as expressed student interest. 

**Table 1:** Lectures included in the Gender and Sexuality in Medicine lecture series with student attendance.

**Year 1: 2018-2019**	Attendance
Birth Control and Family Planning	96
Geriatric and Palliative Care Approach of the LGBTQ Population	23
Perspectives from Intersex Patients	54
HIV Prevention: PEP and PrEP	51
Female Genital Cutting	60
Care for the Transgender Adolescent	70
Callen-Lorde: Innovative practices designed for an LGBT health center	52
Elective Termination of Pregnancy and Miscarriage Management	39
**Year 2: 2019-2020**	
Birth Control and Family Planning	46
Puberty Suppression in Transgender Children	41
Working with Victims of Domestic Violence & Human Trafficking during COVID-19	53
Breast cancer and HPV stigma in immigrant populations	62
STDs and stigma	58
Trauma and PTSD	60

251 medical students attended at least one lecture in the series. 152 (17.7%) students completed the pre-lecture series survey and 105 students completed the post-lecture series survey. Of the 105 (12.3%) medical students who completed the pre and post series surveys, 84 (80%) identify as female and 21 (20%) identify as male. No students identified as a gender minority (transgender or gender nonconforming). 86 (82%) students identify as heterosexual, 19 (18%) identify as sexual minorities, and 1 (1%) preferred not to say. 57 (54%) students identify as White, 23 (22%) identify as Asian, 14 (13%) identify as Black/African American, 6 (6%) identify as Hispanic/Latino, 3 (3%) identify as two or more races/ethnicities, and 2 (2%) identify as other/ preferred not to say (Table 2). These racial/ethnic demographics are similar to that of the overall medical student body at New York Medical College, in which 49% identify as White, 26% identify as Asian, 6% identify as Black/African American, 9% identify as Hispanic/Latino, and 10% identify as other/ preferred not to say. 

**Table 2:** Demographic characteristics of students who completed the pre and post lecture series surveys.

Student Demographics	Pre-test [N (%)]	Post-test [N (%)]
**Year of Participation**	152	105
2018-2019	63 (41.4)	59 (56)
2019-2020	89 (58.6)	46 (44)
**Gender**		
Woman	118 (77.6)	84 (80)
Man	34 (22.4)	21 (20)
**Sexual Orientation**		
Heterosexual or straight	126 (82.9)	86 (82)
Bisexual	14 (9.2)	8 (8)
Gay	8 (5.3)	8 (8)
Lesbian	2 (1.3)	2 (2)
Prefer not to answer	2 (1.3)	1 (1)
**Expected Graduation Date**		
2019	2 (1.3)	0 (0)
2020	4 (2.6)	2 (2)
2021	40 (26.3)	52 (50)
2022	50 (32.9)	36 (34)
2023	2 (1.3)	15 (14)
**Race/Ethnicity**		
Hispanic or Latino	12 (7.9)	6 (6)
Asian	40 (26.3)	23 (22)
Black or African American	9 (5.9)	14 (13)
White	75 (49.3)	57 (54)
2 or more	7 (4.6)	3 (3)
Other/prefer not to say	9 (5.9)	2 (2)

Compared to pre-lecture series survey responses, post-lecture responses indicated a higher percentage of students were “somewhat” or “very” comfortable discussing sexual health topics with adults, adolescents, trans, and LGB patients (Table 3). Additionally, student responses indicated a higher percentage of students were “somewhat” or “very” comfortable discussing elective termination of pregnancy, sexual violence, contraception, medical transitioning, and HIV Pre-exposure Prophylaxis (PrEP) with patients, and identifying Female Genital Cutting (FGC) on physical exam (Table 3). 

**Table 3:** Student comfort with sexual health content pre and post-completion of the lecture series. Percentage of students “somewhat” or “very” comfortable reported with 95% confidence intervals.

How comfortable are you talking to __ patients about issues related to sexuality?				
	Adult	Adolescent	Trans	LGB
Pre Series	59% [51 , 67]	51% [43 , 59]	29% [22 , 36]	49% [41 , 57]
Post Series	90% [85 , 96]	83% [76 , 90]	68% [59 , 77]	84% [77 , 91]
How comfortable are you discussing __ with patients?				
	Elective Termination of Pregnancy	Sexual Violence	Contraception Options	Medical Transition
Pre Series	51% [43 , 59]	33% [25 , 40]	78% [71 , 84]	22% [15 , 28]
Post Series	73% [65 , 82]	65% [55 , 74]	93% [88 , 98]	57% [48 , 67]
How comfortable are you __?				
	Identifying Female Genital Cutting	Counseling patients on PrEP						
Pre Series	11% [6 , 16]	27% [20 , 34]		
Post Series	44% [34 , 53]	70% [61 , 78]		

Students (n=39) provided qualitative feedback on the strengths of the lecture series and areas of the series that could be improved (Table 4). Investigators found that students appreciated the inclusion of relevant material absent from formal medical school curriculum, the focus on underrepresented patient populations, the variety of topics covered by the series, and the depth of material covered by content area experts. In addition, students expressed a desire to expand the series to incorporate more lectures (in total), to increase interactive components of lectures, and for the lecture series to have a more developmental and integrated structure. 

**Table 4:** Themes and illustrative quotes: student perceptions of the strengths and areas of improvement for the Gender and Sexuality in Medicine lecture series (N=39).

Lecture series strengths
Relevance of material absent from formal medical school curriculum.
● “Excellent way to educate future physicians on sensitive topics that are often not well represented in traditional curricula.”
Focus on underrepresented patient populations.
● “This seminar series provided medical students access to a population that is in dire need of attention at this time in America and worldwide.”
Variety of topics covered.
● “Broad range of topics not covered in normal class”
● “Loved the variety of different topics.”
Depth of material and inclusion of clinical pearls by experts in the field.
● “All presenters had extensive experience and it was excellent to hear them speak and give examples”
● “Hearing personal experiences and from experts. It gets you thinking about how you will handle these issues in the clinic and how you can do better at providing care for all”
**Areas of improvement**
Expansion of the number of lectures.
● “I wish that there were more speakers scheduled!”
Increase interactive opportunities.
● “More sessions integrated into the curriculum. Opportunity to practice with standardized patients.”
Increase structure and continuity of lectures.
● “More organization and structure. Perhaps confer with speakers ahead of time about specific sub-topics that would be helpful for the students”

## Discussion

### Clinical and Public Health Implications

The health disparities disproportionately affecting sexual and gender minorities compared to heterosexual and cis-gender individuals are well studied ( 16 ). Emerging research has found that provider bias and inadequate training contribute to these disparities ( 12 , 17 ). Both implicit and explicit discriminatory treatment of GSM patients by medical providers results in healthcare avoidance, which further contributes to worse health outcomes ( 13 , 18 ). Previous studies have shown that many medical students observe anti-GSM sentiment during their medical school training, and that medical school is a critical period for the prevention of healthcare provider bias ( 19 ). GSM-focused curricula may improve physician knowledge of and attitudes towards GSM patients, and stop the cycle of implicit bias in medicine ( 20 , 21 ). Initiatives similar to a student-driven Gender and Sexuality in Medicine lecture series have the potential to mitigate anti-GSM bias in a new generation of physicians. 

It is crucial to bridge the educational gap in reproductive health in medical school curricula in order to reduce major public health consequences for women, including unintended pregnancy Women rely on their physicians for contraception counseling and prevention of unintended pregnancies, yet current medical education provides insufficient coverage of the diverse contraception options available ( 22 ). This study suggests that supplemental lectures significantly improved medical student comfort with providing patients with contraception and elective termination counseling and may serve as a model to address identified training gaps.

Training gaps also exist in the domains of female genital cutting and intimate partner violence. Although over 200 million women and girls worldwide have undergone FGC ( 23 ), there are no established medical school education goals on the topic ( 24 ). Medical education focused on intimate partner violence is also limited ( 7 ). Studies have shown that comprehensive training in FGC and IPV during medical education enhances providers’ abilities to provide quality, compassionate, and culturally sensitive care ( 22 , 25 ). Our student-driven Gender and Sexuality in Medicine lecture series provides an opportunity to improve student comfort in these sensitive topic areas. 

Although Pre-Exposure Prophylaxis (PrEP) can be prescribed by primary care physicians, few primary care providers are comfortable prescribing PrEP compared to their specialist counterparts ( 14 ). Our study found that students who completed the lecture series reported greater comfort counseling patients on PrEP. Previous studies have found that increased comfort discussing STI and HIV prevention with patients is associated with increased provider testing for STIs, and improved patient care ( 26 ). Our study outlines a strategy to help ensure that future providers will be not only comfortable discussing STI prevention but also comfortable testing for the appropriate STIs and counseling patients on PrEP. 

### Importance of Student-Driven GSM Lecture Series

Medical student feedback after attending a student-initiated Gender and Sexuality in Medicine lecture series suggested significantly greater comfort discussing sexual health with adult, adolescent, transgender and LGB-identified patients. Additionally, medical students who attended at least five lectures in the series indicated greater comfort discussing specific topics relevant to the patient care, including elective termination of pregnancy, sexual violence, contraception, medical transitioning, and HIV Pre-exposure prophylaxis (PrEP) with patients, and identifying female genital cutting (FGC) on physical exam. Increasing student comfort with these topics may minimize bias in these future healthcare providers and thus facilitate more informed and comprehensive patient care. 

Overall, our findings are consistent with previously published studies which found that medical students were more confident performing clinical assessments of GSM patients, using appropriate GSM terminology, and were more comfortable providing LGBT-specific care after completing a teaching program specific to LGBT patients ( 27 , 28 ). Though our findings are consistent with these studies, considerable differences exist between our project design compared to previously published studies. Specifically, our lecture series included content highly relevant to, but not limited to, GSM groups. In addition, our study is student-facilitated, and not included as a required component of the medical school curriculum. Though the relative value of mandatory versus non-mandatory medical student sexual education curricula remains to be studied, informal, student-initiated curricula appear to be an essential component of medical student sexual health education ( 10 ). Finally, while there are few studies that evaluate medical student-initiated sexual health curricula specific to LGBT patients, studies show medical student-initiated broad sexual health curricula increase knowledge and willingness to discuss sexual health topics in middle school students ( 29 ). This is consistent with our findings.

### Strengths and Limitations

Despite strong data in support of our student-initiated lecture series, our study does have some limitations. Social desirability bias could influence student self-report of their confidence in sexual health domains. To reduce the likelihood of this bias, all surveys were conducted anonymously to maintain confidentiality. However, because data was collected anonymously, investigators were unable to link specific participant data between pre and post-lecture series datasets. In addition, our survey population was limited to only those students who chose to attend the lecture series. The high overall attendance of first- and second-year students, despite the voluntary nature of our lecture series, demonstrates student interest in these topics, particularly amongst female students. A final limitation of this investigation is that the seminar series was interrupted secondary to the COVID-19 pandemic. This disruption decreased the total number of lectures presented during the academic year, and therefore prevented some students from completing the lecture series, who might otherwise have participated. We have addressed this shortcoming for the 2020-21 academic year by offering lectures virtually. 

In spite of identified weaknesses, our study has many merits. This student-initiated medical education program has demonstrated a positive impact. The incorporation of student input from a variety of perspectives facilitated the inclusion of varied and diverse lecture content material, expanding beyond health consequences among GSM patients. Our qualitative findings demonstrate the importance of showcasing material that is absent from formal medical school curriculum, and student appreciation for in-depth presentation of varied topics from content area experts. 

## Conclusion

Future directions that will be pursued by study investigators include the reformatting of the seminar series onto a virtual platform to make the content accessible to more students. In addition, efforts are underway to increase the variety of topics presented by using needs assessment data. Given the success of this program, we plan to organize data from this investigation and present these findings to the medical school curriculum committee to justify the inclusion of relevant topic areas into the required MD curriculum. 

Our findings demonstrate that student-initiated lecture series can improve medical student comfort discussing sensitive topics related to gender and sexual health with diverse patient populations. This study illustrates the need for innovation in medical education to create more culturally competent and confident physicians, and to better address the health of their future patients. 
